# Adrenal Crisis Caused by Inhaled Fluticasone in an Adolescent with Cystic Fibrosis and Advanced Hepatopathy: A Case Report

**DOI:** 10.1155/2012/913574

**Published:** 2012-09-29

**Authors:** C. Denne, A. E. Vogl-Voswinckel, A. Gruebl, S. Burdach

**Affiliations:** Department of Pediatrics, Kinderklinik München Schwabing, Klinikum Schwabing StKM GmbH and Klinikum Rechts der Isar (AöR) of the Technical University (TU) München, 80804 Munich, Germany

## Abstract

Inhaled corticosteroids are widely accepted in the treatment of cystic fibrosis. Long-term use may cause systemic complications, especially high-dose fluticasone. We report about a young man who presented with encephalopathy after excessive physical activity caused by secondary adrenal insufficiency. He recovered quickly after systemic corticosteroid replacement therapy. This problem is considered to be underdiagnosed in clinical practice.

## 1. Introduction

Inhaled corticosteroids (ICS) control the cystic fibrosis (CF) inherent local pulmonary inflammatory reaction and airway reactivity [1]. ICS given for a long term and in higher doses can cause systemic complications as were cataracts, osteoporosis, and hypothalamic-pituitary-adrenal axis (HPA) suppression because of absorption from the lung and partial clearance at first pass if swallowed [2]. We report about a young CF patient who presented with an acute encephalopathy at the age of 17 years due to secondary adrenal insufficiency caused by fluticasone inhalation therapy.

## 2. Case

**Figure 1:**
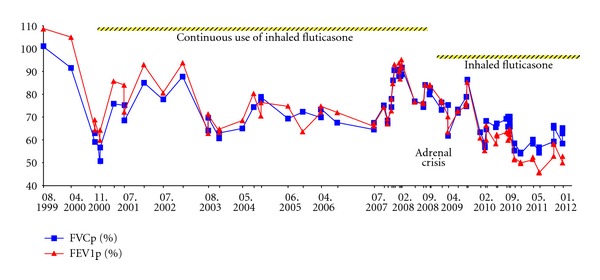
Forced vital capacities and forced expiratory volumes in percent of predicted values (FVCp, FEV1p) from August 1999 until the adrenal crisis in October 2008 show only a slight lung affection of the CF patient. Fluticasone has been prescribed since 1999 in a current dose of 500 *μ*g per day. All visits on which the patient had body plethysmography are listed (month/year). The data after 2008 show the CF disease inherent worsening of lung function.

The patient suffers from an advanced CF-related hepatopathy with grade 2 cirrhosis [4]. It is complicated by coagulation disorder, portal hypertension, esophageal varices (operations in 2007 and 2008), chronic hyperammonemia, and splenomegalia. His lung function remained stable with an inspired vital capacity (VC) of 3.0 L (86% predicted) and a forced expiratory volume in 1 second (FEV1) of 2.5 L (85% predicted). Pseudomonas species is persistently found in his sputum. Furthermore, he is sensitised to Aspergillus (specific immunoglobulin E, IgE, antibody concentration 30.4 kU/L, CAP class 4) and grass pollen (1.78 kU/L, CAP class 2). He has a positive skin prick test for birch. However, he has no clinical apparent seasonal allergic complaints. Combined salmeterol/fluticasone inhalation had been prescribed continuously since he had been 9 years old in a current fluticasone dose of 500 *μ*g per day because of severe bronchial hyperreactivity. He had recurrent airway infections with wheezing and with prolonged cough attacks especially at nights so, doses of 250–1000 *μ*g per day were necessary for symptom control. In the year preceding the adrenal crisis, the adolescent inhaled daily doses of 500–1000 *μ*g. In 2008, after a long bicycle ride the patient felt very exhausted. On the next day, he was too weak to drink, and he vomited several times at night. In the following morning, he was admitted with impaired vigilance and dehydration. He had an ammonia-like oral foetor with Kussmaul respiratory pattern, unclear and slurred speech, severe concentration disorder, and Glasgow Coma Scale [5] (GCS) of 11, but no meningism or flapping tremor. Blood pressure: 82/45 mmHg (<5th percentile for systole [6]), oxygen saturations of 97% on air. *Laboratory data (local lab norm in brackets)*: ammonia 85 *μ*moL/L (<55), standard bicarbonate 18.3 mmoL/L (23–27), base excess −6.6 mmoL/L (from −2 to +2), sodium 125 mmoL/L (134–144), potassium 4.7 mmoL/L (3.4–4.4), International Normalized Ratio (INR) 1.5 (norm 1.0), bilirubin 1.8 mg/dL (<1.1), thyroid stimulating hormone 6.2 mU/L (<4.4), and platelets 73 G/L (150–450). Microbiological examinations were negative. *Electroencephalography* (EEG) showed moderate impairment of brain function with generalized monomorphic theta activity. *Cerebral imaging* could rule out intracranial pathology. In the intensive care unit hyperammonia was treated by arginine-hydrochloride infusions over several hours and the hyponatremia by infusions of physiologic saline solution. Hyperammonia normalized quickly and the encephalopathy resolved completely within 2 days. However, the patient still felt tired. The blood sodium could not be corrected above 130 mmol/L by electrolyte infusions. The patient also presented hypothermia (minimal rectal temperature of 35.8°C) several days after admission. The extended diagnostic work-up to rule out endocrinologic malfunction showed low levels of baseline cortisol and other hormones (Table 1). In the ACTH stimulation test, cortisol remained suppressed. Under the suspicion of a secondary adrenal insufficiency therapy with hydrocortisone (15 mg per square meter body surface per day) and fludrocortisone (0.05 mg bid) was started 10 days after admission. Electrolytes and the clinical state (tiredness, level of activity) improved promptly. Shortly after discharge, bronchial hyperreactivity prompted a restart of ICS (Figure 1). So far, the patient has not shown another episode of a clinical apparent adrenal insufficiency under permanently inhaled fluticasone. Currently (2012), the patient takes 17.5 mg hydrocortisone per day (12.1 mg per square meter BS) in addition to 5 mg prednisolone per day to avoid graft rejection after liver transplantation in 2010.

## 3. Discussion

Adrenal crisis is a life-threatening medical condition with high mortality especially in children and adolescents [7]. Physical exertion may be a disposing factor as cortisol demand rises as in other stress situations [8]. Intravenous fluids are needed to restore intravascular volume as well as a prompt substitution of intravenous corticosteroids. Dehydration may have also contributed to the encephalopathy of the patient. Interestingly, the patient had hyponatremia and hyperkalemia at presentation. Though not typical for secondary adrenal insufficiency, this is to be interpreted as a sign of mineralocorticoid deficiency [9]. The sodium level might also be influenced by an inappropriate antidiuretic hormone secretion, resulting from the loss of physiological inhibition of pituitary vasopressin release by glucocorticoids [10]. Hyponatremic dehydration is also typical for pseudo-Barrter syndrome in CF patients [11]. However, this is exclusively seen in infants and young children, often as an initial first sign of CF. These patients typically show hypokalemia and a pronounced alkalosis which was not the case in our adolescent [12]. Cortisol deficiency by exposure of the HPA axis to exogenous corticosteroids can be a complication of all forms of steroid therapy. Adrenal suppression is more prevalent in long-term therapy and with elevated doses [13]. There are only several cases presenting secondary adrenal insufficiency due to ICS in the literature [14]. German drug data sheets do not recommend higher doses than 1000 *μ*g fluticasone per day for long-term use for children above 16 years of age [15]. There are some hints that fluticasone may already have systemic side effects in doses starting with 200 *μ*g per day in the form of impaired bone growth [16]. However, doses of ≤500 *μ*g of inhaled fluticasone per day are considered safe by several authors [17] due to the low systemic bioavailability compared to older ICS like beclomethasone [18]. At a higher doses however, the high lipophilicity of fluticasone leads to higher tissue concentrations with longer elimination half-life and to accumulation. It has also the longest glucocorticoid-receptor half-life in comparison to other ICS [19]. These specific pharmacokinetics might explain why fluticasone is the most frequent agent found in adrenal failures despite the fact that it is prescribed in lower numbers than concurrent ICS [20].

The CF disease could have been protective against systemic ICS effects. It was shown for fluticasone that absorption is higher in healthy individuals than in asthmatics [21]. Thickened mucus layers and the complex ventilation disorder might correspondingly also reduce systemic absorption in CF patients. As another protective factor, CF patients often demonstrate increased corticosteroid metabolism [22]. However, severe liver disease such as cirrhosis, like in our CF patient, can increase the susceptibility for the development of an adrenal crisis: low cortisol levels are often observed in patients with liver cirrhosis, and hepatic glucocorticoid clearance is impaired by the loss of hepatic enzyme synthesis and liver function [23]. It was demonstrated in a young CF patient that adrenal insufficiency may develop rapidly and at comparatively low ICS doses if the activity of CYP3A4, the major glucocorticoid metabolizing enzyme, is reduced by other drugs such as the antifungal Fluconazole [24]. Besides, his progressive liver failure itself might will have also contributed to the encephalopathy.

## 4. Conclusion

ICS can have systemic effects, namely fluticasone in elevated doses for a long term. Hepatopathy can predispose CF patients to systemic effects as elimination is impaired by a decline of CYP3A4 enzyme functions. Adrenal function loss must be ruled out in every unclear acute cognitive disorder or clouding of consciousness in CF patients under ICS.

## Figures and Tables

**Table 1:** Hormone laboratory values at admission. The low cortisol level 60 minutes after stimulation with 25 IE ACTH 1–24 intravenously is indicative of hypopituitary adrenal insufficiency.

	Result	Reference ranges
*Hormones*		
Baseline cortisol	<1.1 *μ*g/dL	6.0–26.0
Adrenocorticotropine (ACTH)	<5 ng/L	<46
Renin	824.5 ng/L	1.4–17.4
Luteinizing hormone (LH)	4.6 U/L	0.8–7.6
Dehydroepiandrosterone sulfate (DHEAS)	<0.1 *μ*g/mL	0.8–5.6
Follicle-stimulating hormone (FSH)	4.0 U/L	0.7–11.1
Testosterone	13.3 nmol/L	8.0–27.7
*ACTH stimulation test*		
Cortisol after 0 minutes	<1.1 *μ*g/dL	6.0–26.0
Cortisol after 60 minutes	1.9 *μ*g/dL	Threshold values for cortisol peak range from 18.1 to 21.7 *μ*g/dL*

*Petersenn et al. [3].
